# The incidence and etiology of sixth cranial nerve palsy in Koreans: A 10-year nationwide cohort study

**DOI:** 10.1038/s41598-019-54975-5

**Published:** 2019-12-05

**Authors:** Eun Hye Jung, Seong-Joon Kim, Joo Yeon Lee, Bum-Joo Cho

**Affiliations:** 1 Department of Ophthalmology, Nowon Eulji Medical Center, Eulji University, Seoul, Korea; 2 Department of Ophthalmology, Seoul National University College of Medicine, Seoul, Korea; 3 Department of Ophthalmology, Hallym University Sacred Heart Hospital, Hallym University College of Medicine, Anyang, Korea

**Keywords:** Epidemiology, Ocular motility disorders

## Abstract

We aimed to investigate the incidence, prevalence, and etiology of sixth cranial nerve (CN6) palsy in the general Korean population. The nationally representative dataset of the Korea National Health Insurance Service–National Sample Cohort from 2006 through 2015 was analyzed. The incidence and prevalence of CN6 palsy were estimated in the cohort population, confirming that incident cases of CN6 palsy involved a preceding disease-free period of ≥4 years. The etiologies of CN6 palsy were presumed using comorbidity conditions. Among the 1,108,256 cohort subjects, CN6 palsy developed in 486 patients during the 10-year follow-up. The overall incidence of CN6 palsy was estimated to be 4.66 per 100,000 person-years (95% confidence interval [CI], 4.26–5.08) in the general population. This incidence increased with age, accelerating after 60 years of age and peaking at 70–74 years of age. The mean male-to-female incidence ratio was estimated as 1.41 in the whole population, and the incidence and prevalence of CN6 palsy showed an increasing trend over time in the study period. Surgical incidence for CN6 palsy was only 0.19 per 100,000 person-years (95% CI, 0.12–0.29). The etiologies were presumed to be vascular (56.6%), idiopathic (27.2%), neoplastic (5.6%), and traumatic (4.9%). In conclusion, the incidence of CN6 palsy increases with age, peaking at around 70 years, and shows a mild male predominance in Koreans.

## Introduction

Sixth cranial nerve (CN6) palsy, also known as abducens nerve palsy, is a strabismic disorder commonly observed in ophthalmology and neurology clinics and is the most common type of ocular motor nerve palsy^1–3^. Various causes of CN6 palsy, including neoplastic, traumatic, or microvascular diseases, have been reported, some of which are fatal and require further neurological treatment^4–6^.

Thus far, the clinical features of CN6 palsy have mostly been investigated in institution-based studies focused mainly on its causes and prognosis^3,6–10^. Studies on the epidemiology of CN6 palsy using a population-based method are limited^5^. Consequently, the incidence and prevalence of CN6 palsy in the general population remain unknown.

In the present study, we aimed to examine the epidemiology of CN6 palsy in the general Korean population using the Korea National Health Insurance Service–National Sample Cohort (NHIS-NSC) database^11^. We conducted a population-based study to determine the incidence, prevalence, and etiology of CN6 palsy. To our knowledge, this is one of the largest population-based studies on the incidence of CN6 palsy.

## Methods

This cohort study was approved by the Institutional Review Board of Hallym University Medical Center (IRB No. 2018-06-001), which waived the requirement for informed consent. The study complied with the tenets of the Declaration of Helsinki.

### Dataset and study population

The nationally representative NHIS-NSC dataset was used for analyses^11^. The NHIS-NSC of Korea consisted of approximately 1 million participants (2.2% of the Korean population) who were selected by random sampling stratified for age, sex, income, and residential area^11,12^. As for the diagnostic coding, the Korean Classification of Diseases (KCD) system, which is based on the International Classification of Diseases 10^th^ Edition (ICD-10), was adopted in the NHIS-NSC system^11^. Details of the construction and analysis method of the NHIS-NSC are described elsewhere^11^.

We obtained the second version of the NHIS-NSC dataset, which covers the period from 2002 to 2015 and was released in 2017.

### Definition of CN6 palsy and surgeries

**Figure 1:**
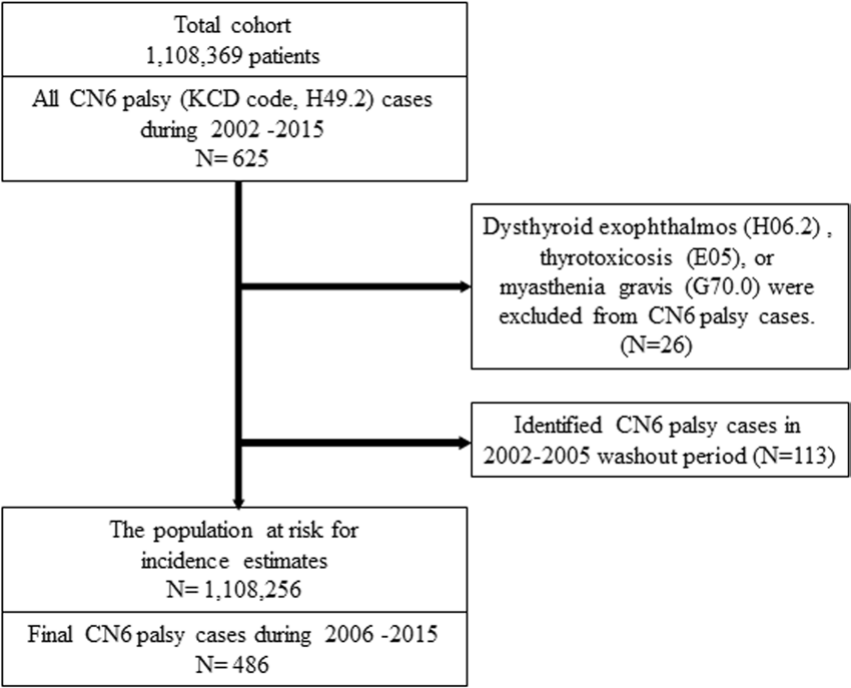
A flowchart of the process to identify newly developed sixth cranial nerve (CN6) palsy.

The participation flowchart of this study is presented in Fig. 1. Patients with CN6 palsy were identified using claims with the KCD code for sixth (abducens) nerve palsy (H49.2) assigned by ophthalmologists, neurologists, or neurosurgeons. As in previous studies, subjects who had comorbid dysthyroid exophthalmos (H06.2), thyrotoxicosis (hyperthyroidism, E05), or myasthenia gravis (G70.0) were excluded in consideration of misdiagnosis^4,13,14^. An incident CN6 palsy case was defined based on the earliest CN6 palsy diagnosis for each subject with a preceding 4-year or longer disease-free period. To secure the preceding 4-year disease-free period, patients diagnosed with CN6 palsy between 2002 and 2005 were excluded.

The incidence and prevalence of CN6 palsy were investigated by sex, age-group, and year of diagnosis. A conservative approach (prevalent cases considered those with clinic visits per calendar year) was used to determine prevalence^15^. The annual incidence and prevalence were estimated using the number of patients who qualified for NHIS-in each year^15^. Surgery for CN6 palsy, defined as strabismus surgeries performed after the diagnosis of CN6 palsy during the follow-up period, was identified using the Korean Electronic Data Interchange codes for strabismus surgeries (S5173, 5174, 5175, or 5176).

### Etiologies of CN6 palsy

Because medical-record details are not available for the NHIS-NSC database, the causes of CN6 palsy were presumed based on the principal diagnostic codes for comorbidities registered in the NHIS-NSC database within 6 months before and 2 weeks after the first diagnosis of CN6 palsy. Table 1 presents the associated diagnostic codes of possible etiologies of CN6 palsy. The etiologies of CN6 palsy considered in the current study included vascular diseases, cerebral aneurysm, trauma, intracranial neoplasm, intracranial inflammation or infection, and others^5^. Aneurysm was categorized separately from the vascular diseases. Subjects without the diseases mentioned in Table 1 were classified as idiopathic cases. If a subject had multiple co-morbidities, the etiology was determined by the consensus of two examiners (EHJ and B-JC).

**Table 1:** Diagnostic codes for possible comorbid etiologies of sixth nerve palsy.

Cardiovascular or Cerebrovascular disease
Hypertension (I10‐15)
Diabetes mellitus (E11‐14)
Ischemic heart diseases (I20–25)
Peripheral vascular disease (I70, I73.1, I73.8, I73.9, I79.2)
Cerebrovascular disease (I60-68, G45, and G46) including stroke (I60, 61, 62, 63, and 64) and transient ischemic attack (G45.8, G45.9), and except aneurysm(I67.1)
**Cerebral aneurysm (I67.1)**
Trauma
Fracture of skull and facial bones (S02.0, S02.1, S02.3, S02.4, S02.7, S02.8)
Contusion of eyeball and orbital tissues (S05.1)
Intracranial injury (S06)
Neoplasm
Malignant neoplasm of eye and adnexa (C69)
Malignant neoplasm of meninges (C70)
Malignant neoplasm of brain (C71)
Malignant neoplasm of spinal cord, cranial nerves and other parts of central nervous system (C72)
Secondary malignant neoplasm of brain and cerebral meninges (C79.3)
Malignant neoplasm of nasopharynx (C11)
Malignant neoplasm of craniofacial bones (C41.00)
Benign neoplasm of meninges (D32)
Benign neoplasm of brain and other parts of central nervous system (D33)
Neoplasm of uncertain or unknown behavior of meninges (D42)
Neoplasm of uncertain or unknown behavior of brain and central nervous system (D43)
Hemangioma of intracranial structures (D18.01)
Inflammation or infection
Inflammatory diseases of the central nervous system(G00-G07)
Viral infections of the central nervous system(A81-A89)
Cysticercosis of central nervous system (B69.0)
Others
Multiple sclerosis (G35)
Guillain-Barré syndrome (G61.0)
Sarcoidosis (D86)
Hydrocephalus (G91)
Benign intracranial hypertension (G93.2)
Arachnoid cyst (G93.0)
Inflammation or disorder of orbit (H05.0, H05.1, H05.8, H05.9)

### Statistical analysis

The incidence and prevalence were estimated with a 95% confidence interval (CI) based on a Poisson distribution. All statistical analyses were performed using SAS Enterprise Guide version 7.1 (SAS Institute, Cary, NC, USA) and R version 3.4.3 (The R Foundation for Statistical Computing, Vienna, Austria).

## Results

A total of 1,108,369 subjects were included in the cohort (Fig. 1). Among these, 625 individuals were diagnosed with CN6 palsy at least once from 2002 to 2015. Of these, 26 subjects who had comorbid thyrotoxicosis or myasthenia gravis were excluded. An additional 113 subjects with a history of CN6 palsy from 2002 to 2005 were also excluded. Finally, 486 individuals were identified as having newly developed CN6 palsy during the 10-year follow-up period. Among them, 285 (58.6%) were males and 201 (41.4%) were females.

### Incidence of CN6 palsy

**Figure 2:**
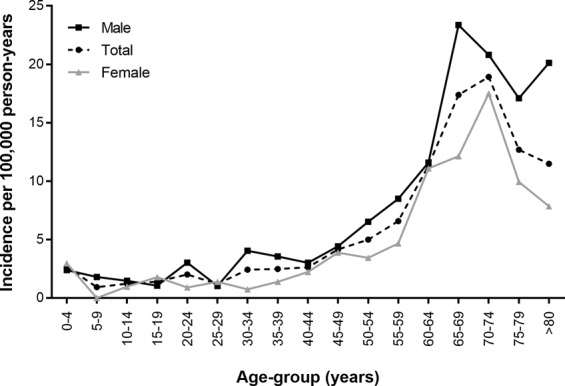
Incidence of sixth cranial nerve palsy by age group.

Table 2 and Fig. 2 show the incidence of CN6 palsy according to age-group and sex. The estimated overall incidence of CN6 palsy in the entire cohort was 4.66 (95% CI, 4.26–5.08) per 100,000 person-years. The incidence of CN6 palsy was 5.45 (95% CI, 4.84–6.11) per 100,000 person-years for males and 3.86 (95% CI, 3.35–4.42) per 100,000 person-years for females. The overall incidence of CN6 palsy increased with age, with peaks at 65–69 years of age in males and at 70–74 years of age in females.

**Table 2:** Incidence of sixth cranial nerve palsy in the general Korean population from 2006 to 2015.

Age (years)	Total				Male				Female				MF ratio	Surgery
	Person-years	N	Incidence	95% CI	Person-years	N	Incidence	95% CI	Person-years	N	Incidence	95% CI		N
0–4	486198	13	2.67	1.47, 4.40	250727	6	2.39	0.95, 4.85	235470	7	2.97	1.28, 5.75	0.80	2
5–9	535292	5	0.93	0.33, 2.01	278148	5	1.80	0.64, 3.86	257143	0	0.00	NA	NA	2
10–14	647077	8	1.24	0.56, 2.30	339623	5	1.47	0.53, 3.16	307454	3	0.98	0.24, 2.53	1.51	0
15–19	709306	10	1.41	0.71, 2.47	374819	4	1.07	0.33, 2.48	334488	6	1.79	0.71, 3.63	0.59	0
20–24	691976	14	2.02	1.14, 3.28	363632	11	3.03	1.57, 5.18	328345	3	0.91	0.23, 2.37	3.31	0
25–29	746079	9	1.21	0.58, 2.17	386066	4	1.04	0.32, 2.41	360013	5	1.39	0.50, 2.98	0.75	2
30–34	821811	20	2.43	1.52, 3.66	420206	17	4.05	2.41, 6.28	401605	3	0.75	0.19, 1.94	5.42	0
35–39	882081	22	2.49	1.59, 3.69	448967	16	3.56	2.09, 5.61	433114	6	1.39	0.55, 2.81	2.57	1
40–44	910933	24	2.63	1.72, 3.83	463826	14	3.02	1.70, 4.89	447107	10	2.24	1.12, 3.92	1.35	0
45–49	889115	37	4.16	2.96, 5.65	451548	20	4.43	2.76, 6.66	437566	17	3.89	2.32, 6.04	1.14	5
50–54	820034	41	5.00	3.62, 6.69	413348	27	6.53	4.37, 9.31	406686	14	3.44	1.94, 5.57	1.90	1
55–59	637981	42	6.58	4.79, 8.78	317471	27	8.50	5.69, 12.13	320511	15	4.68	2.69, 7.46	1.82	2
60–64	476515	54	11.33	8.57, 14.63	232976	27	11.59	7.75, 16.53	243539	27	11.09	7.41, 15.81	1.05	1
65–69	402543	70	17.39	13.63, 21.79	188243	44	23.37	17.13, 30.98	214302	26	12.13	8.05, 17.41	1.93	2
70–74	332766	63	18.93	14.63, 24.00	144176	30	20.81	14.22, 29.17	188591	33	17.50	12.19, 24.17	1.19	1
75–79	228505	29	12.69	8.61, 17.89	87694	15	17.10	9.84, 27.28	140812	14	9.94	5.60, 16.10	1.72	0
>80	217542	25	11.49	7.56, 16.60	64599	13	20.12	11.07, 33.13	152943	12	7.85	4.20, 13.16	2.56	1
Overall	10435755	486	4.66	4.26, 5.08	5226069	285	5.45	4.84, 6.11	5209686	201	3.86	3.35, 4.42	1.41	20

**Figure 3:**
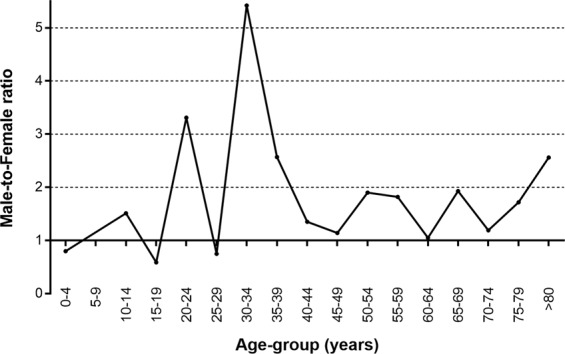
Male-to-female ratio for the incidence of sixth cranial nerve palsy.

Figure 3 shows the male-to-female ratio of the incidence of CN6 palsy according to age-group. The incidence of CN6 palsy was slightly higher in males than in females, and the average male-to-female incidence ratio was 1.41. Male preponderance was highest in the group of subjects 30–34 years of age and was consistent throughout the cohort, except for the groups of subjects 15–19 and 25–29 years of age.

**Figure 4:**
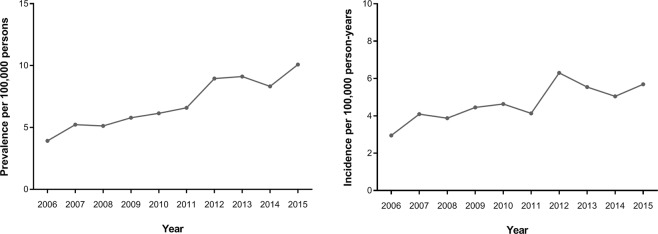
Annual prevalence (Left) and incidence (Right) of sixth cranial nerve palsy.

The annual incidence of CN6 palsy grossly increased over time in the study period, as presented in Table 3 and Fig. 4. Of the 486 subjects diagnosed with CN6 palsy from 2006 to 2015, 20 underwent strabismus surgeries. The incidence of surgery for CN6 palsy in the general population was 0.19 per 100,000 person-years (95% CI, 0.12–0.29).

**Table 3:** Annual prevalence and incidence of sixth cranial nerve palsy in the general Korean population from 2006 to 2015.

Year	Prevalence											Incidence			
	Population	Total			Male			Female			Surgery	Person-years	No.	Incidence	95% CI
		No.	Prevalence	95% CI	No.	Prevalence	95% CI	No.	Prevalence	95% CI	No.				
2006	1021208	40	3.92	2.83, 5.26	23	4.49	2.90, 6.59	17	3.34	1.99, 5.18	4	1018279	30	2.95	2.01, 4.13
2007	1031653	54	5.23	3.96, 6.76	29	5.61	3.81, 7.90	25	4.86	3.19, 7.02	3	1026145	42	4.09	2.98, 5.46
2008	1035089	53	5.12	3.86, 6.63	24	4.63	3.01, 6.73	29	5.62	3.81, 7.91	0	1032300	40	3.87	2.79, 5.20
2009	1038462	60	5.78	4.44, 7.37	37	7.11	5.05, 9.66	23	4.44	2.86, 6.51	4	1032802	46	4.45	3.29,5.87
2010	1042706	64	6.14	4.75, 7.77	32	6.13	4.24, 8.50	32	6.15	4.26, 8.53	1	1036952	48	4.63	3.44, 6.06
2011	1046465	69	6.59	5.16, 8.27	36	6.87	4.86, 9.36	33	6.32	4.40, 8.72	3	1040580	43	4.13	3.02, 5.49
2012	1050743	94	8.95	7.26, 10.88	57	10.84	8.26, 13.90	37	7.05	5.02, 9.57	2	1047571	66	6.30	4.90, 7.94
2013	1053952	96	9.11	7.41, 11.05	52	9.86	7.42, 12.79	44	8.36	6.12, 11.08	6	1047853	58	5.54	4.23, 7.08
2014	1057454	88	8.32	6.70, 10.18	50	9.45	7.07, 12.32	38	7.19	5.14, 9.73	2	1051316	53	5.04	3.80, 6.52
2015	1061141	107	10.08	8.29, 12.12	54	10.17	7.70, 13.13	52	9.80	7.38, 12.72	3	1054824	60	5.69	4.37, 7.25

### Prevalence of CN6 palsy

Table 3 and Fig. 4 also present the annual prevalence rates of CN6 palsy during the period from 2006 to 2015. The prevalence of CN6 palsy was 3.92 (95% CI, 2.83–5.26) per 100,000 persons in 2006, but gradually increased, reaching 10.08 (95% CI, 8.29–12.12) per 100,000 persons in 2015. There was an overall increasing trend in the prevalence of CN6 palsy over time (Fig. 4). The annual prevalence of CN6 palsy was slightly higher in males than in females, except in 2008 and 2010.

### Etiologies of CN6 palsy

**Figure 5:**
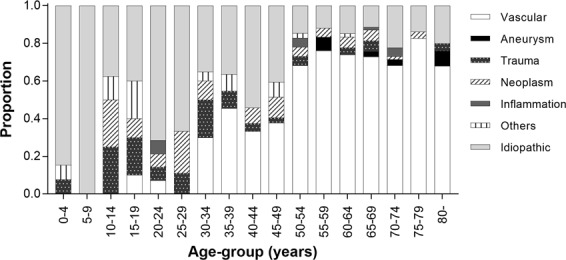
Etiologies of sixth cranial nerve palsy by age groups in Koreans.

Etiologies of CN6 palsy were evaluated in 486 subjects in 2006–2015. The most common etiology was presumed to be vascular disease (n = 275, 56.6%), followed by idiopathic (n = 132, 27.2%), intracranial neoplasm (n = 27, 5.6%), trauma (n = 24, 4.9%), cerebral aneurysm (n = 9, 1.9%), and intracranial inflammation or infection (n = 7, 1.4%). The etiologies of 12 subjects (2.5%) were classified as “other”; these included multiple sclerosis (n = 1), Guillain-Barré syndrome (n = 4), benign intracranial hypertension (n = 1), arachnoid cyst (n = 3), and inflammation or disorder of orbit (n = 3). The etiologies of CN6 palsy according to age group are presented in Fig. 5. In subjects aged ≥50 years, most of the etiologies were presumed to be vascular disease (n- = 235, 72.5%). There were 24 subjects with head trauma, which was most common among young adults aged 30–39 years (n = 6, 25.0%).

Surgeries for CN6 palsy were performed in subjects with idiopathic causes (n = 9, 6.8% of idiopathic cases), vascular disease (n = 6, 2.2%), intracranial neoplasm (n = 3, 11.1%), cerebral aneurysm (n = 1, 11.1%), and arachnoid cyst (n = 1, 33.3%).

## Discussion

In this population-based cohort study, the overall incidence of CN6 palsy in Korea was 4.66 per 100,000 person-years; the incidence rate increased with age, accelerating after the age of 60 years and peaking between 70–74 years of age. An increasing trend in the overall incidence and prevalence of CN6 palsy was observed during the study period. The most common presumed etiology was vascular disease, but the etiologies did vary by age-group.

In the present study, we found that the incidence of CN6 palsy in Koreans is slightly lower than that in Americans reported by Patel *et al*. (4.66 vs. 11.3 per 100,000 person-years), although the age-distribution and peak incidence of CN6 palsy were similar^5^. Of note, Repka *et al*. reported that strabismus is less common in Asian races than in the white race^16^; however, the differences in paralytic strabismus among different ethnicities have not been widely reported. The difference of the epidemiology of CN6 palsy among different races needs to be studied further. In addition, we also observed overall increasing trends in both the annual prevalences and incidences of CN6 palsy over time.

Results of the current study revealed a mild male preponderance of CN6 palsy in Koreans and an overall male-to-female ratio of 1.41. This phenomenon has been observed in several previous studies, with male-to-female ratios ranging from 1.2 to 1.8^3,6,17^. Although some studies reported no sex predilection for CN6 palsy^18^, one of the suggested explanations for male preponderance of CN6 palsy is the higher frequency of head trauma in males than in females^19^. In the present study, male preponderance was prominent in the group of subjects aged 30–34 years, and the age group with the highest frequency of head trauma was also young adults aged 30–39 years. Another possible explanation could be that male gender is a risk factor for diabetes or hypertension in Korea^20,21^. The higher risk of microvascular disease in male might be associated with the preponderance of CN6 palsy, especially in older age groups.

Studies on the etiology of CN6 palsy have reported high frequencies of microvascular disease (28–46%)^3,5,18^ or unknown origin (24–31%)^3–5,10,13,18^. It is widely reported that microvascular diseases are a common cause of isolated unilateral CN6 palsy in patients over 50 years of age^1^. The association between age and incidence in the present study might be related to the age-dependent increase of vasculopathic diseases, such as hypertension, diabetes, or other cerebrovascular diseases. We also observed that vascular disease was the most common cause of CN6 palsy, and the proportion was higher than in previous results of up to 39%. This variety in the etiology distribution may be attributed to variable inclusion criteria for vascular causes. We considered not only diabetes and hypertension but also other cerebro- or cardiovascular diseases as vascular factors. Tamhankar *et al*. also reported that CN6 palsy in 80.6% of patients over 50 years of age was due to microvascular causes^2^.

Although neuroimaging techniques have improved, the rate of idiopathic CN6 palsy still remains high; recent rates are not lower than rates reported from the 1950s through the 1980s^1,3,5^. On the other hand, the proportion of aneurysmal origin in CN6 palsy is low (0–6%) in the past and recent studies^4,5,8,13,14,18,22,23^. Studies on CN6 palsy in a series of children reported a high frequency of neoplastic etiology (39–45%)^8,23^. Similarly, a high proportion of neoplasm was also observed in the group of subjects aged 10–19 years in the present study.

Recovery rates of CN6 palsy have been reported as high as 60.0 to 87.3%^3,10,24^. Vascular and idiopathic etiologies were associated with higher natural recovery rates than other etiologies of ocular motor nerve palsies^3,4,9,22^. Sanders *et al*. reported that 51 of 59 patients (86%) experienced resolution of CN6 palsy, and only 3 patients required strabismus surgery^17^. Peragallo *et al*. reported that 4% of patients underwent strabismus procedures, and etiologies of surgeries for CN6 palsy included neoplastic (22%), traumatic (27%), idiopathic/microvascular (28%), and other causes (22%)^25^. In our study, the incidence of surgery for CN6 palsy was also rare. The main causes of CN6 palsy requiring surgery was vascular or idiopathic as in the whole patients with CN6 palsy, and this was due to the high number of vascular and idiopathic groups.

There were a few potential sources of bias and limitations in this study. First, the incidence and prevalence may have been underestimated, as subjects who did not visit the hospitals were missed. In addition, the diagnosis of CN6 palsy was confirmed using only the national health insurance database, not by detailed ophthalmological medical records, because full medical records are not available in the claim database. Nevertheless, the diagnosis of CN6 palsy is usually straightforward, and the chance of misdiagnosis is relatively low. As such, many studies on paralytic strabismus have used the NHIS-NSC dataset^26,27^. Lastly, the causal relationships remain unclear, because the etiologies were attributed to comorbid diseases associated with the CN6 palsy diagnosis.

In conclusion, we found the incidence of CN6 palsy in Korea to be 4.66 per 100,000 person-years. It increased with age, especially after the age of 60 years. The most common cause of CN6 palsy was presumed to be vascular disease in the general population, but the etiologies varied according to age group. Clarification of the epidemiology of CN6 palsy may help us to understand the pathophysiology of the disorder and to build public policies for its prevention and treatment.
